# Impact of Earthquakes During COVID-19 Lockdown on the Pediatric Injury Pattern in the Zagreb Urban Area

**DOI:** 10.3390/jcm14020640

**Published:** 2025-01-20

**Authors:** Dino Bobovec, Tomislav Žigman, Josip Lovaković, Goran Augustin, Anko Antabak, Ivan Dobrić

**Affiliations:** 1 Department of Surgery, University Hospital Centre Zagreb, Kišpatićeva 12, 10000 Zagreb, Croatia; dino.bobovec@kbc-zagreb.hr (D.B.); tomislav.zigman@kbc-zagreb.hr (T.Ž.); josip.lovakovic@kbc-zagreb.hr (J.L.); anko.antabak@kbc-zagreb.hr (A.A.); ivan.dobric@kbc-zagreb.hr (I.D.); 2 School of Medicine, University of Zagreb, Šalata 2, 10000 Zagreb, Croatia

**Keywords:** wound, injury, child, COVID-19, earthquake

## Abstract

**Background/Objectives**: Previous works on the epidemiology of pediatric trauma during the COVID-19 lockdown observed a decrease in pediatric surgical emergency consultations and fracture referrals. None of those works describes a unique situation in which there is the coexistence of another opposing factor, like an earthquake, that influences the number of injured children’s referrals. Therefore, this study aimed to investigate the influence of earthquakes during the COVID-19 lockdown on pediatric injury pattern referrals at a tertiary care hospital in a urban setting. **Methods**: A retrospective single-center case-control study comprised a time interval at the time of the COVID-19 lockdown, starting with a day when the biggest earthquake happened and finishing at the end of the confinement period in Zagreb, Croatia (22 March–27 April 2020). The control group comprised the identical time interval in 2019. We identified all successive pediatric trauma patients referred to the Pediatric Emergency Department. Demographics and leading injury characteristics were analyzed. **Results**: We analyzed data from 1166 patients. In the case group, the median age was lower than in the control group but without gender differences. We detected a decrease in Pediatric Emergency Department referrals and a reduced proportion of pediatric trauma patients in the case group. Additionally, the proportion of shoulder/elbow injuries and head injuries was higher, and the proportion of foot/ankle injuries was lower in the case period than in the control period. **Conclusions**: Earthquakes during the COVID-19 lockdown changed the pattern of pediatric injuries. These data can be used to restructure health resources during similar conditions to provide optimal health care to children.

## 1. Introduction

To confront the coronavirus disease 2019 (COVID-19), governments and healthcare systems implemented different strategies worldwide. Croatia’s healthcare system, which is universal and primarily public, with better accessibility to healthcare facilities in major cities, had to reorganize rapidly to optimize its resource allocation. As proposed by many scientific societies, vast non-emergency surgical activity was suspended and priority groups for malignancies were defined [1,2]. The Croatian government imposed a lockdown from 19 March until 27 April 2020, restricting children to their homes and indoor activities. Consequently, the risk factors and mechanisms of injuries were altered, since pediatric trauma is mainly associated with sports and outdoor activities during warm weather [3,4,5,6]. During the lockdown in Croatia, on 22 March 2020, a series of earthquakes happened in Zagreb. The largest earthquake had a maximal macroseismic intensity of VII–VIII according to the Medvedev–Sponheuer–Karnikova scale and a magnitude (ML) of 5.5. One child was killed, 26 people were injured, and 16,555 residential, public and monumental buildings were declared damaged or destroyed [7]. Although some studies have tried to determine the influence of the lockdown on pediatric trauma emergencies, none of the published works that describe pediatric trauma describe a simultaneous natural disaster during the studied period [8,9,10,11,12,13,14,15,16,17,18]. Moreover, none of the works describes a unique situation in which two opposing factors influence the number of injured children’s referrals (i.e., earthquake increases, whereas lockdown reduces the number of injured children referrals) [19,20,21,22,23,24,25]. Therefore, since the coexisting natural disaster during a COVID-19 lockdown presents a unique state of emergency yet unfamiliar to the literature, this study aimed to investigate the influence of earthquakes at the time of the COVID-19 lockdown on pediatric injury pattern referrals at a tertiary care hospital in an urban setting.

## 2. Materials and Methods

A retrospective single-center case-control study was performed after institutional review board approval (No: 02/21 AG Class: 8.1–20/153-2) at the University Hospital Centre Zagreb. We investigated two intervals. The case group included a time interval during the COVID-19 lockdown, starting with the day when the biggest earthquake happened and finishing at the end of the lockdown in Zagreb (22 March–27 April 2020). The control group corresponded to the identical time interval in 2019 (22 March–27 April 2019). The study included all consecutive pediatric trauma patients (soft tissue, musculoskeletal, or visceral) who presented to the Pediatric Emergency Department. Patients above the age of 18 years and readmissions to the hospital were excluded. The variables collected were patient age, sex, type, and region of leading injury. Leading injury types were divided into four major categories: contusions, sprains, joint dislocations, and fractures. Furthermore, we divided the anatomic regions of the leading injury into five main groups: upper and lower limbs, spine/pelvis, thorax/abdomen, and head. The main groups were later subdivided into 18 anatomic groups: head, spine, thorax, abdomen, pelvis, hip, thigh, knee, lower leg, ankle, foot, clavicle, shoulder, upper arm, elbow, forearm, wrist, and hand. All groups were analyzed separately. Also, patients admitted to the hospital due to urgent surgical care (e.g., elastic stable intramedullary nail, open/closed reduction, and fixation with Kirschner wires) were investigated independently in both time intervals.

A median (range) was used to report the continuous variables. A count (percent) was used to present the categorical variables. A Mann–Whitney U test and a Chi-square test were used to analyze two intervals. To compare two proportions, we used a two-proportions Z-test. The significance level for all *p* values was <0.05. Statistical analyses were performed using MedCalc, version 16.4.3 (MedCalc Software bv, Ostend, Belgium) [26].

## 3. Results

We identified 1465 pediatric surgical patients who presented to the Pediatric Emergency Department in the observed period. A total of 1166 patients met the inclusion criteria and were further analyzed.

The overall median age of the studied groups was 11 (0–18) years, and 37.7% (440/1166) were girls. The median age of the case group was lower than that of the control group (8 vs. 11 years, U = 1167.5, z = −2.67508, *p* = 0.00736) but without statistically significant sex differences. The demographic data of each group are reported in Table 1.

In the case group, we detected a reduction in pediatric trauma referrals (225 vs. 941; *p* ≤0.0001) with a decreased proportion of pediatric trauma presentations within the total pediatric surgical emergency presentations (74.5% vs. 80.9%; z = 2.4618, *p* = 0.0139). The types of leading injuries are grouped in Table 2.

In the case group, we detected a significant increase in the proportion of head injuries (29.8% vs 18.2%, z = −3.8801, *p* = 0.0001) and shoulder/elbow region injuries (13.3% vs. 5.5%, z = −4.1145, *p* ≤ 0.00001) in comparison to the control group. On the contrary, in the case group, there was a significant decrease in the proportion of foot/ankle region injuries (8% vs. 21.7%, z = 4.6949, *p* < 0.00001) and a trend toward a decrease in the proportion of knee injuries (3.6% vs. 7.1%, z = 1.9579, *p* = 0.05), although without statistical significance. A detailed regional pattern of leading injuries is reported in Table 3.

The proportion of hospital admissions (5.5% vs. 6.6%, *p* = 0.50926) and the need for surgical treatment (2.3% vs. 3.9%, *p* = 0.08186) remained similar in both intervals.

## 4. Discussion

Earthquakes during the COVID-19 lockdown influenced the pediatric injury referral pattern at a tertiary care facility in the Zagreb urban area. Upper extremity trauma remained the most common region of pediatric injury in both cohorts.

Since a coexisting natural disaster during a COVID-19 lockdown presents a unique state of emergency yet unfamiliar to the literature, this study aimed to determine the effect of earthquakes in the time of a COVID-19 lockdown on the injury pattern in children. Therefore, to prevent hospital overcrowding and resource deficiency, which could subsequently decrease patient care, it is important to know which injuries are infrequent and which appear more frequently during natural disasters, especially because public health systems are responsible for the continuum of diagnosis and treatment of all patients. Such data can be useful in crisis management when planning the initial response to future natural disasters, e.g., redistribution of the surgical workforce and resources to meet the expected change in surgical workload.

Analysis of the patients’ demographic data revealed no connection between sex and the state of the emergency (Table 1). On the other hand, the median age of the case group was lower than the control group, which agrees with the data from studies investigating the impact of the lockdown and COVID-19 pandemic on pediatric surgical referrals [19,20,21]. Some authors point out the suspension of all sporting activities during the lockdown as a possible explanation for the observed age shift, since sport was previously identified as a leading cause of trauma in the adolescent age group [4,20]. Moreover, during the post-earthquake panic, younger children are more prone to be struck by or against objects (i.e., walking into a wall, a closed door, furniture, or debris).

In the case group, we observed a significant reduction in total pediatric surgical emergency referrals, with a decreased proportion of pediatric trauma referrals within the pediatric surgical emergency referrals. This is in accordance with previous works on the epidemiology of pediatric trauma during the COVID-19 lockdown, which observed a decrease in total pediatric surgical emergency consultations and a 2.5-fold decrease in fracture referrals attributed to a substantial reduction in playground and sports accidents [20,21]. This finding may also indicate that only the more severely injured children were referred to the hospital because of transportation problems (e.g., public road traffic restrictions due to the curfew, post-earthquake debris on the roads, or damaged cars).

Furthermore, we observed no change in the proportion of trauma cases that required hospitalization or surgical intervention. This must be observed in the context of a substantial reduction of pediatric trauma referrals in the case period. In contrast, it would be reasonable to expect that the number of seriously injured children (i.e., ones that required hospitalization and/or surgical intervention) would be higher in the case period. Following that, we may conclude that the number of seriously injured children decreased in the case period, which is in concordance with the current literature [8].

The lower number of severely injured children in the case period may also indicate that children during earthquakes are less likely to get hurt due to their smaller volume since they can adapt to the limited free area under the debris. Conversely, it may point out a higher on-site mortality [25].

Upper limb trauma remains the most common region of pediatric injury despite coexisting lockdowns and natural disasters. Moreover, we observed an injury pattern shift during the case period since there was a significantly higher proportion of head and shoulder/elbow injuries. Also, a significantly lower proportion of foot/ankle injuries was observed in the case period, with a trend toward a decrease in the proportion of knee injuries. This is in accordance with the work on pediatric injury epidemiology during lockdown, which observed a predominance of upper extremity injuries during the pre-pandemic and lockdown periods [21]. Additionally, those results partially oppose the previous works on both pediatric and adult earthquake victims, which indicate that lower limb and head injuries were most common among surviving victims [22,23,24,25,26]. The predominance of lower limb and head injuries during earthquakes is likely because the injury mechanisms differ from everyday life (e.g., impact against collapsing walls or falling debris versus falling onto an outstretched hand) [24].

We may presume that the decreased frequency of lower limb injuries in the case group is due to lockdown, which decreased the average running, jumping, and pivoting time by ceasing sporting and playground activities, thus lowering the danger of ankle torsions and falls [20]. This presumption is supported by a significantly lower proportion of sprains in the case group (Table 2).

The limitations of this study are its relatively short investigation time, retrospective single-center design, and the specific local healthcare system organization. Moreover, different governments imposed different lockdown types, which may have influenced the results. Also, since earthquakes and lockdowns coexisted, it could be hard to differentiate whether the change in the injury pattern is from lockdowns, earthquakes, or both, which limits the study results’ generalizability. To overcome this, we propose that further research should be muti-center with a design to control potential confounding bias better. However, our results may predict a similar scenario in future crises.

## 5. Conclusions

In our case-control retrospective study evaluating the pediatric injury patterns presenting to the emergency department during the COVID-19 lockdown and associated with earthquakes, we noted a significant change in emergency room visits, with upper extremity injuries remaining the most common type. These data can be used to restructure health resources during similar conditions to provide optimal healthcare to children. Moreover, to make these data more generalizable, we propose that further research be multi-center with a design that would control potential confounding bias.

## Figures and Tables

**Table 1:** Demographical data in two periods.

Demographical Data	Case Group	Control Group
Total number of patients	225	941
Age, median, *n*	8	11
Age, mean, *n*	8.2	10.2
Female *n* (%)	88 (39.1%)	352 (37.4%)

**Table 2:** Types of leading injury in two periods.

Type of Leading Injury	Case Group (225 Patients), *n* (%)	Control Group (941 Patients), *n* (%)	*p*-Value
Contusion	140 (62.2%)	555 (59%)	0.3734
Sprain	9 (4%)	120 (12.8%)	0.0001
Joint dislocation	7 (3.1%)	18 (1.9%)	0.267
Fracture	68 (30.2%)	244 (25.9%)	0.1902
Laceration	0 (0%)	3 (0.3%)	0.3953
Burn	1 (0.4%)	1 (0.1%)	0.2713

**Table 3:** Anatomic groups of leading injury regions in two periods.

Leading Injury Region	Case Group (225 Patients), *n* (%)	Control Group (941 Patients), *n* (%)	*p*-Value
Head	67 (29.8%)	171 (18.2%)	0.0001
Thorax/abdomen	1 (0.4%)	21 (2.2%)	0.0767
Thorax	1 (0.4%)	11 (1.2%)	0.3320
Abdomen	0 (0%)	10 (1%)	0.1211
Spine/pelvis	4 (1.8%)	32 (3.4%)	0.2076
Spine	3 (1.3%)	26 (2.8%)	0.2149
Pelvis	1 (0.4%)	6 (0.6%)	0.7278
Upper limb	116 (51.6%)	416 (44.2%)	0.0466
Clavicle	5 (2.2%)	8 (0.9%)	0.0784
Shoulder	4 (1.8%)	11 (1.2%)	0.4654
Upper arm	12 (5.3%)	11 (1.2%)	<0.00001
Elbow	14 (6.2%)	30 (3.2%)	0.0315
Forearm	14 (6.2%)	48 (5.1%)	0.5028
Wrist	30 (13.3%)	97 (10.3%)	0.1902
Hand	37 (16.4%)	211 (22.4%)	0.0488
Lower limb	37 (16.4%)	301 (32%)	<0.00001
Hip	1 (0.4%)	1 (0.1%)	0.2713
Thigh	3 (1.3%)	11 (1.2%)	0.8414
Knee	8 (3.6%)	67 (7.1%)	0.05
Lower leg	7 (3.1%)	18 (1.9%)	0.267
Ankle	10 (4.4%)	120 (12.7%)	0.0003
Foot	8 (3.6%)	84 (8.9%)	0.0073

## Data Availability

The raw data supporting the conclusions of this article will be made available by the authors on request.
